# Polyphenols for improvement of inflammation and symptoms in rheumatic diseases: systematic review

**DOI:** 10.1590/1516-3180.2020.0766.R1.22042021

**Published:** 2021-11-15

**Authors:** Hillary Nascimento Coletro, Amanda Popolino Diniz, Nathália Sernizon Guimarães, Júlia Cristina Cardoso Carraro, Raquel de Deus Mendonça, Adriana Lúcia Meireles

**Affiliations:** I MSc. Doctoral Student, Postgraduate Program on Health and Nutrition, School of Nutrition, Universidade Federal de Ouro Preto (UFOP), Ouro Preto (MG), Brazil; Doctoral Student, Grupo de Pesquisa e Ensino em Nutrição e Saúde Coletiva (GPENSC), Ouro Preto (MG), Brazil.; II MSc. Doctoral Student, Postgraduate Program on Health and Nutrition, School of Nutrition, Universidade Federal de Ouro Preto (UFOP), Ouro Preto (MG), Brazil; Doctoral Student, Grupo de Pesquisa e Ensino em Nutrição e Saúde Coletiva (GPENSC), Ouro Preto (MG), Brazil.; III PhD. Former Postdoctoral Fellow in the Postgraduate Program on Health and Nutrition, School of Nutrition, Universidade Federal de Ouro Preto (UFOP), Ouro Preto (MG), Brazil.; IV PhD. Adjunct Professor, Department of Clinical and Social Nutrition, Universidade Federal de Ouro Preto (UFOP), Ouro Preto (MG), Brazil; Adjunct Professor, Grupo de Pesquisa e Ensino em Nutrição e Saúde Coletiva (GPENSC), Ouro Preto (MG), Brazil.; V PhD. Adjunct Professor, Department of Clinical and Social Nutrition, School of Nutrition, Universidade Federal de Ouro Preto (UFOP), Ouro Preto (MG), Brazil; Adjunct Professor, Grupo de Pesquisa e Ensino em Nutrição e Saúde Coletiva (GPENSC), Ouro Preto (MG), Brazil.; VI PhD. Adjunct Professor, Department of Clinical and Social Nutrition, School of Nutrition, Universidade Federal de Ouro Preto (UFOP), Ouro Preto (MG), Brazil; Adjunct Professor, Grupo de Pesquisa e Ensino em Nutrição e Saúde Coletiva (GPENSC), Ouro Preto (MG), Brazil.

**Keywords:** Antioxidants, Rheumatic diseases, Polyphenols, Rheumatoid arthritis, Osteoarthritis, Phenolic compounds, Nutrition, Epidemiology, Human health

## Abstract

**BACKGROUND:**

Rheumatic diseases (RDs) are a group of pathological conditions characterized by inflammation and functional disability. There is evidence suggesting that regular consumption of polyphenols has therapeutic effects capable of relieving RD symptoms.

**OBJECTIVE:**

To synthesize data from randomized controlled trials on administration of polyphenols and their effects on RD activity.

**DESIGN AND SETTING:**

Systematic review conducted at Universidade Federal de Ouro Preto, Minas Gerais, Brazil.

**METHODS:**

A systematic search was conducted in the databases PubMed (Medline), LILACS (BVS), IBECS (BVS), CUMED (BVS), BINACIS (BVS), EMBASE, Web of Science and Cochrane Library and in the grey literature. The present study followed a PRISMA-P checklist.

**RESULTS:**

In total, 646 articles were considered potentially eligible, of which 33 were then subjected to complete reading. Out of these, 17 randomized controlled trials articles were selected to form the final sample. Among these 17 articles, 64.71% assessed osteoarthritis (n = 11), 23.53% rheumatoid arthritis (n = 4), 5.88% rheumatoid arthritis and fibromyalgia (n = 1) and 5.88% osteoarthritis and rheumatoid (n = 1). Intake of polyphenol showed positive effects in most of the studies assessed (94.12%): it improved pain (64.70%) and inflammation (58.82%).

**CONCLUSION:**

Polyphenols are potential allies for treating RD activity. However, the range of polyphenol sources administered was a limitation of this review, as also was the lack of information about the methodological characteristics of the studies evaluated. Thus, further primary studies are needed in order to evaluate the effects of polyphenol consumption for reducing RD activity.

**SYSTEMATIC REVIEW REGISTER:**

PROSPERO - CRD42020145349.

## INTRODUCTION

Rheumatic diseases (RDs) belong to a group of chronic musculoskeletal pathological conditions characterized by joint damage, inflammation, pain, functional disability and impact on individuals’ quality of life.^1–4^ Rheumatic diseases include chronic clinical conditions of multicausal etiopathogenesis characterized by disruption of immunological tolerance, production of autoantibodies, production of a number of substances accounting for lesions in many body structures,^4^ mechanical stress in the joints and changes to the alignment of bones, cartilage and other structures necessary for joint stability.^5^

They give rise to a heterogeneous group of clinical conditions, such as rheumatoid arthritis, osteoarthritis, scleroderma, systemic sclerosis, ankylosing spondylitis, fibromyalgia, osteoporosis, tendinitis, gout and lupus, among others. Osteoarthritis (OA) and rheumatoid arthritis (RA) are the most common RDs.^3^ OA is the first chronic, inflammatory and degenerative disease that arises through joint cartilage wear or loss.^2^ RA is an inflammatory disease that mainly affects joints without being degenerative. Instead, it causes structural damage and joint inflammation, which result in progressive structural and functional losses.^4^

RDs are more common in developed countries and in women. In Europe and North America, their prevalence is 0.5%-1.0%.^6^ OA is the most common form of arthritis, affecting approximately 14 million people in the United States.^7^ Moreover, RA affects about five in every 1,000 adults and the number of individuals affected is expected to rise to approximately 67 million by 2030.^8^

Many treatments for diminishing RD activity are available. These include tumor necrosis factor-alpha inhibitors and the disease-modifying anti-rheumatic drugs (DMARDs) infliximab, etanercept, certolizumab pegol, golimumab, adalimumab, tocilizumab, abatacept, rituximab, tofacitinib, baricitinib, upadacitinib, secukinumab, ustekinumab, ixekizumab, guselkumab and belimumab. However, these can be costly and can have side effects like abdominal pain, back pain, chest pain and nausea.^8^

Diet therapy can be used to assist RD therapies, since it helps to reduce pain and inflammation effects.^9^ The Nurses’ Health Study cohort showed lower RA incidence among individuals who followed healthy dietary patterns (as assessed using the Healthy Eating Index, HEI-2010) than among individuals who followed inadequate dietary patterns.^10^ Regular consumption of fresh fruits, vegetables and spices rich in phytochemicals can mitigate oxidative stress and inflammation, and relieve symptoms.^11^

The therapeutic effects of phytochemicals, especially polyphenols, on RDs have been studied, given their antioxidant, anti-inflammatory and immunomodulatory properties.^3^ Polyphenols are metabolites found in plants that are produced in metabolic pathways triggered by plant interactions with environmental factors. They are involved in plant reproduction and in communication between plants, as well as in their defense against pathogens. Polyphenols are found in vegetables, fruits, cocoa and nuts, and also in their derivatives, such as juices and teas.^12^

Epidemiological studies have presented associations between polyphenol intake and RDs,^13–15^ and experimental studies on animal models and in vitro investigations about the role played by polyphenols in RDs have been conducted. Diets rich in bioactive compounds are associated with improvement of disease activity, since these substances act as protective factors against inflammatory processes and against endothelial dysfunction linked to development of worsening of clinical signs and symptoms.^3^

A systematic review of the literature showed that total flavonoids and specific subclasses of flavonoids such as flavanols, flavanones, flavones, isoflavones and anthocyanins (but without addressing total polyphenols in diets) are associated with low risk of developing diabetes, cardiovascular events and mortality.^16^ However, to the best of our knowledge, no systematic review has been conducted with the aim of evaluating the association between administration of polyphenols and RD symptoms.

## OBJECTIVE

The aim of the present article was to review the effects of polyphenols on RD activity, based on information available in the literature (randomized clinical trials).

## METHODS

### Protocol and registration

The present systematic review was conducted in accordance with the “Preferred Reporting Items for Systematic Reviews and Meta-Analysis” guidelines (PRISMA-P). To define the research question, the PICOS (Patient-Intervention-Comparison-Outcome) strategy was used, as shown in **Supplementary Material Table 1** (available from https://drive.google.com/file/d/106nzdLxTUbI7rRQt9s0kAsA_V8rOvxe6/view?usp=sharing). The analytic methods and inclusion criteria for the present review were documented in a systematic review protocol recorded on the PROSPERO platform of the University of York, United Kingdom (CRD42020145349).

### Information sources

A search was conducted in the PubMed (via Medline), LILACS, IBECS, CUMED, BINACIS, EMBASE, Cochrane Library and Web of Science databases and in the grey literature to find studies in which the aim had been to investigate associations between polyphenol administration and rheumatic diseases. This search was conducted between July 22, 2019, and September 10, 2020.

The descriptors used were previously defined in the MeSH, DECS and Emtree databases. These related to “Rheumatic Diseases” or “Disease, Rheumatic” or “Rheumatic Disease” or “Rheumatism” and “Polyphenols” or “Provinols”. Detailed search strategies are presented in **Supplementary Material** that is available from https://drive.google.com/file/d/106nzdLxTUbI7rRQt9s0kAsA_V8rOvxe6/view?usp=sharing.

### Inclusion and exclusion criteria

Only double-blind randomized controlled trials (RCTs) analyzing outcomes from interventions consisting of polyphenol administration to improve disease activity were included in this study. No restrictions on the date of publication or language used were imposed in relation to article selection.

The exclusion criteria encompassed duplicates, in vitro studies, reviews, cross-sectional or observational studies, case reports, case series, ecological studies, studies about other morbidities or studies on pregnant women, children or teenagers.

### Data collection process

The references retrieved through the search strategies were exported to an Endnote file (Clarivate Analytics, Philadelphia, United States), and duplicates were removed. Two independent researchers (HNC and APD) selected titles and abstracts; potential texts were evaluated to check their eligibility based on the criteria described above. A third researcher (NSG) resolved any discrepancies resulting from disagreements between HNC and APD. In addition, the grey literature, such as monographs, dissertations, theses and conference proceedings, was assessed based on references in the articles selected.

### Data extraction

Two independent researchers (HNC and APD) extracted data on features such as study design, name of the first author, publication year, participants, participants’ age and sex, intervention features, placebo groups (sample, age, sex), intervention types (polyphenol use), sample size and outcomes (rheumatic disease activity: pain, functional capacity, inflammatory markers, laboratory markers, antioxidant activity and quality of life).

### Evaluation of the methodological quality of the studies included

The quality of the RCT methodology was assessed through the Cochrane tool for risk of bias in Cochrane randomized studies (RoB 2.0, London, England), which classifies studies as having high or low risk of bias. The methodological quality was assessed by two independent researchers (HNC and APD), and a third researcher (NSG) resolved any score divergences.

## RESULTS

**Figure 1:**
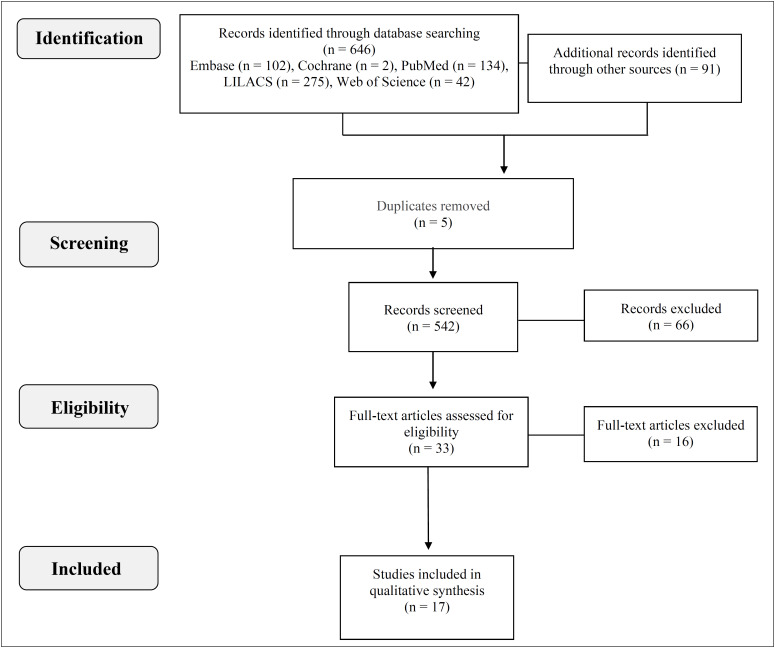
PRISMA flow of studies included in the review.

The search in the databases and in the grey literature resulted in 646 studies. In total, 641 publications were evaluated after duplicate removal (n = 5). From among these, 542 articles were excluded from the sample because of the title and 66 through reading the abstract. The remaining 33 studies were then read in full (Figure 1). Sixteen articles were excluded during this text analysis stage due to their methodologies (pilot studies or experimental studies). Thus, 17 articles composed the final sample of the present review (Figure 1).

Among these 17 clinical trials, 52.94% (n = 9) were published in the last five years (2015-2019); 23.53% were conducted in the United States (n = 4), 17.65% in Iran (n = 3), 11.65% in India (n = 2), 5.89% in Iraq (n = 1), 5.89% in Armenia (n = 1), 5.89% in Australia (n = 1), 5.89% in Japan (n = 1), 5.89% in Egypt (n = 1), 5.89% in Belgium (n = 1), 5.89% in Brazil (n = 1) and 5.89% in Finland (n = 1). The number of individuals evaluated reached 1,244 (the minimum and maximum sample groups encompassed 17 and 201 subjects, respectively). Studies reporting the sample characteristics (n = 14) showed that 55.55% of the participants were women (n = 691). In all the studies, the total of 1,244 participants were in the age group 45-85 years.

The analysis on the studies included in this review was demonstrated through three tables that were organized according to the pathological conditions found. Table 1 presents the results found for studies that assessed OA; Table 2, RA; and Table 3, the studies that assessed both of these diseases plus fibromyalgia (rheumatoid arthritis and fibromyalgia; osteoarthritis and rheumatoid arthritis).

**Table 1:** Features of randomized controlled trials evaluating the effect of polyphenol administration on osteoarthritis

Author	Country, year	Sample features	Intervention	Control group	Outcome	Results after intervention
Du et al.^28^	United States, 2019	n = 49; ♀ = 71.4%; mean age = 55.6 years	40 g/day of blueberry concentrate	Yes	Pain, inflammation and daily performance	↓ stiffness, pain and difficulty performing daily activities (P < 0.05)
						↑ IL-13 in the intervention group (P < 0.05)
						↓ MCP-1 in the intervention group (P < 0.05)
Hussain et al.^21^	Iraq, 2018	n = 92; age group: 45 - 75 years	500 mg/day of resveratrol + 15 mg/day of meloxicam	Yes	Pain and functional disability	↓ stiffness, pain and difficulty performing daily activities (P < 0.05)
Schell et al.^18^	United States, 2017	n = 17; ♀ = 76.5%; mean age = 57 ± 7 years	50 g/day of strawberry concentrate	Yes	Pain, inflammation and quality of life	↓ IL-6, IL-1β and MMP-3 (P < 0.05)
						↓ intermittent pain and constant pain
Wong et al.^20^	Australia, 2017	n = 72; ♀ = 100%; mean age = 61.5 ± 0.9 years	75 mg/day of trans-resveratrol	Yes	Pain, sleep disorders, symptoms of menopause, symptoms of depression, quality of life and mood	Pain improvement/mitigation
						(P = 0.004)
Panahi et al.^33^	Iran, 2015	n = 40; ♂ = 100%; age < 80 years	1500 mg/day of curcuminoid + 15 mg/day of biopterin	Yes	Antioxidant activity and GSH and MDA concentrations	↑ SOD (P < 0.001)
						↓ MDA concentration (P < 0.04)
Shep et al.^22^	India, 2019	n = 139; ♂ = 66.9%	500 mg/day of curcumin	No	Pain	↓ VAS scoring for pain (P < 0.01)
						KOOS score improvement (P < 0.01)
Haroyan et al.^25^	Armenia, 2018	n = 201; ♀ = 93.03%; mean age = 56.2 years	500 mg/day of Curamin and/or 500 mg/day of Curamed	Yes	Pain, inflammation and physical performance	↓ pain parameters (P < 0.01)
						Improved physical and functional performance (P < 0.05)
Nakagawa et al.^32^	Japan, 2014	n = 41; ♀ = 78%; mean age = 68.7 years	6 capsules of Theracurmin/day	Yes	Pain and stiffness, daily performance and health conditions	↓ VAS score for pain (P = 0.023)
Henrotin et al.^17^	Belgium, 2014	n = 22; ♀ = 68.2%; mean age = 64.3 ± 8.4 years	6 capsules of Flexofytol/day	No	Pain and disease activity	↓ biomarker Coll2-1 (P = 0.002)
						↓ disease activity (P = 0.0047)
Naderi et al.^27^	Iran, 2012	n = 100; ♀ = 90% Age group: 50 - 70 years	1 g/day of ginger	Yes	Inflammation	↓ CRP and nitric oxide (P < 0.001)
Shumacher et al.^30^	United States, 2013	n = 58; ♂= 75.86%; mean age = 57 ± 11 years	500 ml of tart cherry juice/day	Yes	Pain, stiffness and functional capacity	↑ WOMAC Score (P = 0.002) Pain improvement (P = 0.042) Functional capacity improvement (P < 0.001) ↓ hsCRP (P = 0.006)

VAS = visual analogue scale; KOOS = Knee Injury and Osteoarthritis Outcome Score; MDA = malondialdehyde; IL = interleukin; MCP-1 = monocyte chemoattractant protein-1; CRP = C-reactive protein; MMP-3 = matrix metalloproteinase-3; M1 = δ-(3,4-dihydroxy-phenyl) – γ- valerolactone; SOD = superoxide dismutase; Coll2-1 = cartilage biomarker; WOMAC = Western Ontario and McMaster Universities Osteoarthritis Index; hsCRP = high-sensitivity C-reactive protein.

**Table 2:** Features of randomized controlled trials evaluating the effect of polyphenol administration on rheumatoid arthritis

Author	Country, year	Sample features	Intervention	Control group	Outcome	Results after intervention
Khojah et al.^23^	Egypt, 2018	n = 100; ♀ = 68%	1 g/day of resveratrol + antirheumatic drugs	Yes	Biochemical and inflammatory markers	↓ CRP (P < 0.05)
						↓ESR, ucOC, MMP-3, TNF-α and IL-6 (P < 0.001)
Chandran and Goel^24^	India, 2012	n = 45; ♀ = 84.4%; mean age = 47.8 years	500 mg/day of curcumin + 50 mg/day of sodium diclofenac	Yes	Disease activity	Mitigating disease activity (P < 0.05)
						↓ CRP (P < 0.05)
Javadi et al.^19^	Iran, 2014	n = 40; ♀ = 100%; mean age = 47.3 years	500 mg/day of quercetin	Yes	Antioxidant capacity	There was no statistically significant difference after intervention with quercetin
Thimotéo et al.^26^	Brazil, 2018	n = 41; ♀ = 100%; mean age = 52.75 years	500 ml of reduced-energy cranberry juice/day	Yes	Biochemical markers and disease activity	Mitigating disease activity (P = 0.048) ↓ anti-CCP (P = 0.034)

ESR = erythrocyte sedimentation rate; ucOC = undercarboxylated osteocalcin; CRP = C-reactive protein; MMP-3 = matrix metalloproteinase-3; IL = interleukin; TNF = tumor necrosis factor; Anti-CCP = anti-cyclic citrullinated peptide.

**Table 3:** Features of randomized controlled trials evaluating the effect of polyphenol administration on rheumatic diseases

Author	Country, year	Sample features	Intervention	Control group	Rheumatic disease	Outcome	Results after intervention
Hänninen, et al.^31^	Finland, 2000	115	Diet of living food*	Yes	Rheumatoid arthritis and fibromyalgia	Symptoms of rheumatoid arthritis and fibromyalgia	↓ rheumatoid arthritis activity (P = 0.03)
							Joint stiffness mitigation (P = 0.001)
							Pain mitigation (P = 0.003)
Bitler, et al.^29^	United States, 2007	90	400 mg/day of olive pulp	Yes	Osteoarthritis and rheumatoid arthritis	Ability to perform daily activities, disease activity and inflammation	↓ CRP (P < 0.01)

* Diet of living food consisted of vegan diet without cooking; CRP = C-reactive protein.

Among RDs, 64.71% of the studies assessed osteoarthritis (n = 11) (Table 1); 23.53%, rheumatoid arthritis (n = 4) (Table 2); 5.88%, rheumatoid arthritis and fibromyalgia; and 5.88%, osteoarthritis and rheumatoid (Table 3).

Polyphenols were administered in the form of either capsules of a specific polyphenol or concentrates of food sources of polyphenols (**Online Supplementary Material**, available from: https://drive.google.com/file/d/106nzdLxTUbI7rRQt9s0kAsA_V8rOvxe6/view?usp=sharing). The polyphenol doses administered ranged from 42 mg/day^17^ to 1,585 mg/day.^18^ **Table 2** in **Online Supplementary Material** (available from https://drive.google.com/file/d/106nzdLxTUbI7rRQt9s0kAsA_V8rOvxe6/view?usp=sharing) describes the doses administered, their source and the polyphenol composition.

The intervention with polyphenols to mitigate/rule out disease activity (pain, functional capacity, inflammatory markers, laboratory markers, antioxidant activity and quality of life) of rheumatic diseases (outcome variable) led to positive results in 94.12% of the studies selected. Pain improved in 64.70% of the cases (n = 11), based on assessment using a visual analogue scale, and inflammation improved in 58.82% of the cases (n = 10). The only study that recorded negative results for RD mitigation only assessed participants’ antioxidant capacity based on biochemical markers.^19^

**Figure 2:**
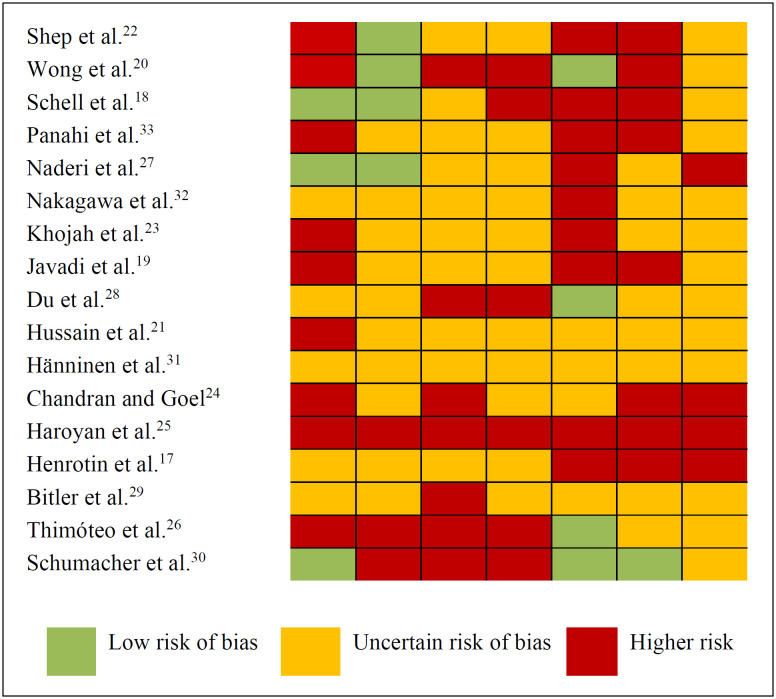
Assessment of the quality of randomized clinical trials selected to form part of the present review, 2020.

The lack of information about the methodological characteristics of the studies evaluated in the present review made it difficult to classify the quality of evidence, as shown in Figure 2. Eight studies did not mention any method of randomization.^19–26^ Among all the articles, five did not mention the allocation method.^18,19,22,23,27^ Nine studies had a high risk of bias because the study participants were not blinded to either the intervention or the placebo groups.^20,21,24,25,29,30^ In six studies, an imbalance in either the number of or the reasons for missing data, between the experimental and control groups, was observed.^18,20,25,26,28,30^ Lastly, just four authors described all the outcomes targeted and measured.^20,26,28,30^

## DISCUSSION

We found out that polyphenols are capable of helping to treat RDs, with reductions of inflammation and pain. Therefore, their use in treatments for RDs can impact the quality of life of the individuals affected.

To the best of our knowledge, the present systematic review was a pioneer in assessing the association between polyphenol administration and mitigation/improvement of rheumatic disease activity in humans.

Positive effects from polyphenol intake on the improvement/mitigation of rheumatic disease activity were observed in most of the studies selected (94.12% of the articles).^17,18,20–33^ Based on the information in these articles, pain and inflammation in patients with osteoarthritis or rheumatoid arthritis were the main symptoms mitigated/relieved in the populations assessed.^17,18,20,21,22,28,32^

The studies showed positive results in terms of reduction of RD activity, due to pain relief;^18,20,21,22,28,30,31,32^ reductions of the levels of cytokines and pro-inflammatory markers such as C-reactive protein (CRP), erythrocyte sedimentation rate (ESR), interleukins 6 (IL-6) and 1β (IL-1β) and tumor necrosis factor α (TNF-α);^18,23,27,29,30^ reductions of disease activity as assessed through reductions of the levels of undercarboxylated osteocalcin (ucOc), matrix metalloproteinases (MMP-3), anti-cyclic citrullinated peptide (anti-CCP) and Coll2-1 markers;^17,18,23,26^ increased levels of anti-inflammatory cytokines, such as IL-13;^28^ and improvements in oxidative stress caused by increasing the levels of antioxidant enzymes such as superoxide dismutase (SOD) and reducing malondialdehyde (MDA).^33^

RD improvement was mostly identified by means of biochemical markers that indicate normal or pathological functioning.^34^ Inflammatory biomarker levels are increased in RDs, and are associated with the pain and other symptoms of the disease.^6^ They can be divided into the following categories: pro-inflammatory cytokines, anti-inflammatory cytokines, adipokines and chemokines. Pro-inflammatory cytokines are mainly produced by adipocytes: the main ones are IL-6, IL-8, IL-1β and TNF-α.^34^ Specific biomarkers of RD, such as MMP, stand out among them. These biomarkers belong to a family of enzymes that account for the extracellular degradation of cartilage matrix components, including collagen type II and aggrecan; they change bone metabolism, cartilage and the synovial membrane, which leads to joint destruction.^35^

There is a specific treatment for each clinical condition in RDs. These treatments can range from medication to secondary therapies such as individualized diet therapy.^6,36^ Overall, drug therapy involves use of non-steroidal anti-inflammatory drugs (NSAIDs) such as diclofenac and meloxicam, but these substances lead to several side effects like peptic ulcers. Accordingly, anti-inflammatory compounds can come from food. Thus, it is essential to define the compounds capable of mitigating pain and inflammation.

Polyphenols have been described in the literature as potent anti-inflammatory drugs that can be used to minimize the effects of diseases on different health conditions.^36^ Polyphenols link to aromatic rings that reduce free radicals, inhibit formation of reactive species during metabolism, perform anti-inflammatory immunomodulatory actions^3^ and have an anabolic effect on cartilage cells.^37^ Experimental studies have already shown the beneficial action of flavonoids with regard to increasing cartilage anabolic activity and improving the levels of insulin-like growth factor-1 (IGF-1), osteocalcin and physical morphogenetic protein.^38^ Reproduced clinical trials have shown that blueberries are a source of polyphenols that have anti-inflammatory effects and can improve gait capacity parameters among older adults.^39,40^

The magnitude of the results recorded can change depending on polyphenol type, dose (extract, fruit concentrate or others), delivery route (oral or injection into the synovial fluid), association with other compounds (such as drug therapy) and the types of markers analyzed.^17^

Polyphenols from different sources were administered in the studies reviewed here. This made it difficult to interpret the results, since there may have different mechanisms of action^41^ and even different degrees of bioavailability.^42–45^ However, studies that have reviewed the effects of polyphenols for prevention or treatment of several diseases used a wide variety of sources and different quantities of polyphenols,^46–48^ given the heterogeneity of sources of polyphenols and the scarcity of existing literature on this subject from primary studies. Lack of information about the medications or dietary supplements used by participants in the 17 studies evaluated may have been another form of bias. Some studies did not mention the type or dosage of medication administered to control the diseases assessed. Therefore, doubts regarding the effects of polyphenols in isolation are raised.

Lack of clarity about several aspects of the studies evaluated in the present review made it difficult to classify the quality of evidence found in these studies. According to Gordis et al.,^49^ randomized controlled clinical trials presenting good methodological quality are characterized by clear planning, execution and reporting, and should guarantee adequate confidentiality of allocation, degree of blinding and randomization. Thus, when these studies are meticulously designed, executed and reported, they can be considered to represent the gold standard for assessing the effectiveness of healthcare interventions. However, despite the large numbers of studies included in the search and analysis processes of the present review, it was not possible to perform a meta-analysis. This was because the studies selected assessed different evaluation parameters for disease activity in RDs, and also used different doses and types of polyphenols.

Given the lack of consensus on the best doses and types of polyphenols in the studies assessed in this review, the results should be interpreted with caution and attention. There is a need to conduct primary studies that focus on the minimum dose necessary to achieve the protective effects of polyphenols on the health of patients with RDs. Accordingly, for better guidance for healthcare professionals and patients, future research must focus on, and align with, daily recommendations for foods that are known to be source of polyphenols that are capable of preventing and protecting health and helping in treating RDs, due to the importance of consuming such bioactive compounds.

Despite the bias in the primary data sources that is reported here, this review produced promising results, considering that, overall, the dietary intake from polyphenol-rich sources had positive effects with regard to reducing both inflammation and the symptoms of RDs.
